# Supplementary material for: Protogyny in a tropical damselfish: females queue for future benefit
Source: PeerJ. 2016 Jun 30;4:e2198. doi: 10.7717/peerj.2198 (PMC4933090; doi:10.7717/peerj.2198)
Supplement: Supplemental Information 1 [file peerj-04-2198-s001.pdf]

McCormick MI - Protogyny in a tropical damselfish: females queue for future benefits

Supplementary files

Gender-specific differences in morphology

Thirteen female, 12 male and 7 immature male (no detectable testes) *P. amboinensis* were collected during the breeding season from the Lizard island fringing reef using clove oil and a hand net. Fish were placed into clipseal plastic bags underwater and photographed laterally against a 1 cm grid to determine their size and morphology. Fish were then taken back to the laboratory, euthanized with a clove-oil overdose and their sex was validated through dissection. Seven morphological variables were measured from the images using Image-J software (Fig. S1). A principal component analysis on the correlation matrix of morphological variables was used to explore the variability in morphology with respect to gender. Eigenvectors illustrated the original morphological variables responsible for the difference between genders.

The analysis indicates that males (and immature males) can be discriminated from females because they are larger, have longer abdomens, deeper in the body at the vent, but shorter keel depth, and have larger dorsal fins (Fig. S2).

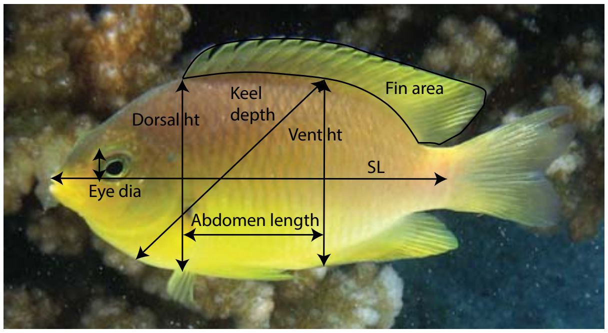

Figure S1. Morphological measurements made in the comparison of morphology among female, immature male and mature male *Pomacentrus amboinensis*.

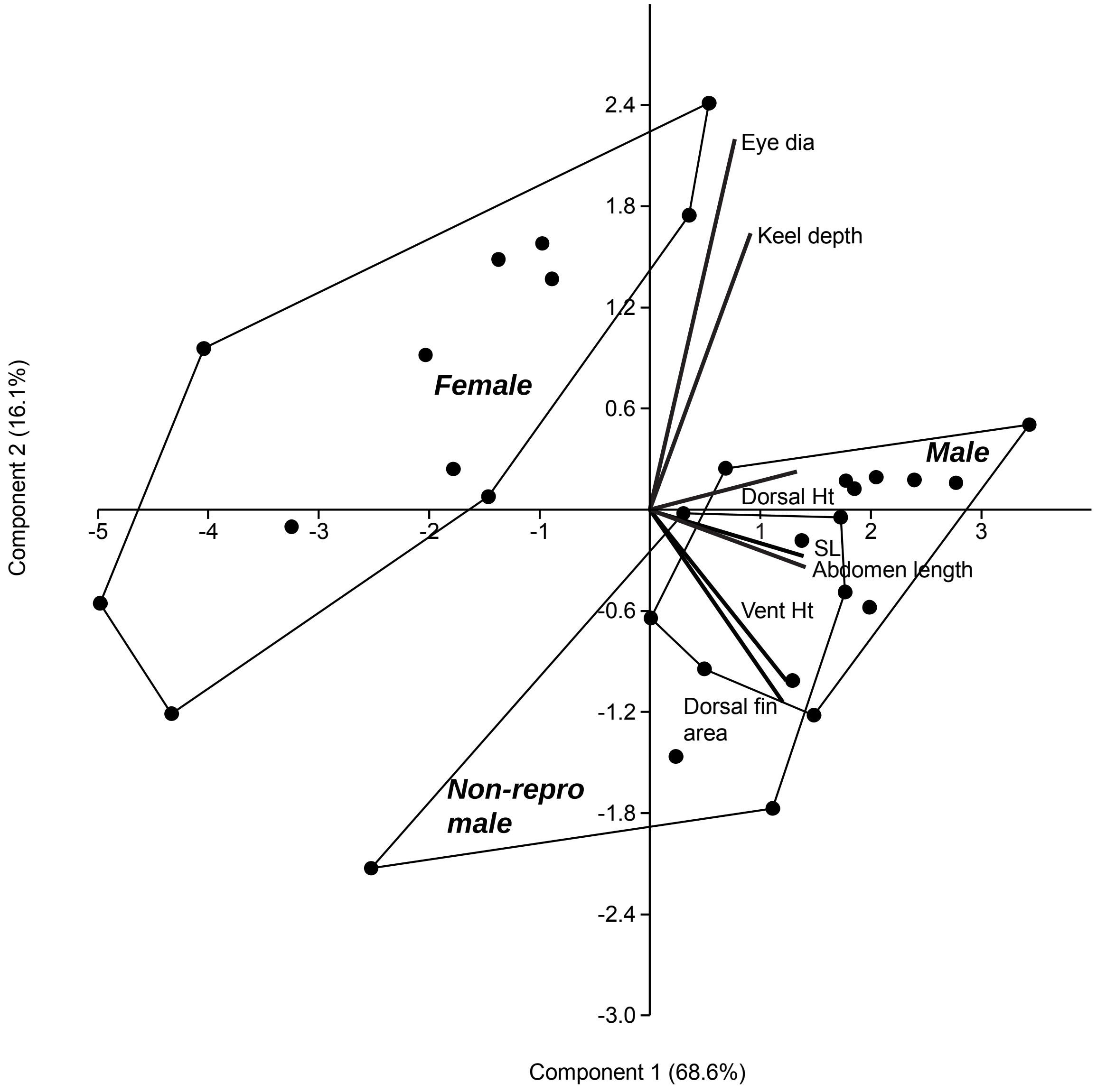

Figure S2. Principal component analysis showing the difference in morphology between male and females. Convex hulls envelope the males, females and non-reproductive males. The morphological variables that best differentiate gender are dorsal fin area, abdominal length, vent height and standard length.

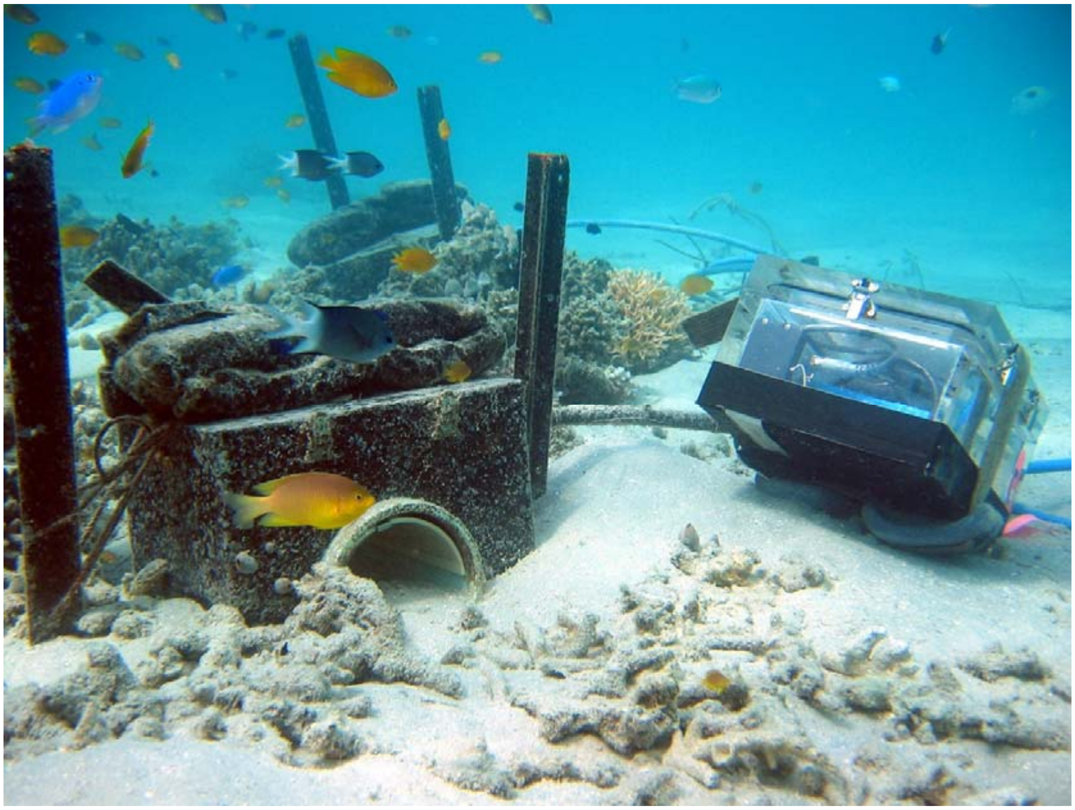

Figure S3. A male *Pomacentrus amboinensis* guards his artificial nest site. Placement of the passive integrated transponder (PIT) tag reader over the nest site, together with the battery and data-logger housing are shown.

Male gut samples

During October to December 1994 the spawning output of 10 reproductive pairs of the *P. amboinensis* was monitored around the Lizard Island fringing reef at each of 5 locations (see McCormick 1998). Natural nests (e.g., upturned clam shells) were replaced with an artificial nesting surface, which consisted of an upturned terracotta roof tile. These tiles presented a uniform concave nesting surface that were of similar dimension and defensibility as natural nests. Nests were monitored for new embryos daily and when found the nests were carefully turned over, a transparent 1cm^2 grid was placed over the top, and the clutch was photographed underwater. The nests were then replaced. The number of embryos were later counted from these photographs. At the end of the 6-week monitoring study, the male and α-female associated with each nest were collected. Gut samples were examined for the presence of eggs. Of the 47 nest-guarding males collected, 8 contained eggs. Those that contained eggs had stomachs that were almost completely full of eggs. Eggs were identified as being fish eggs, most likely

belonging to *P. amboinensis* through microscopic examination. Data suggests that it is generally the more reproductively successful fish that engaged in filial cannibalism (Fig. S4).

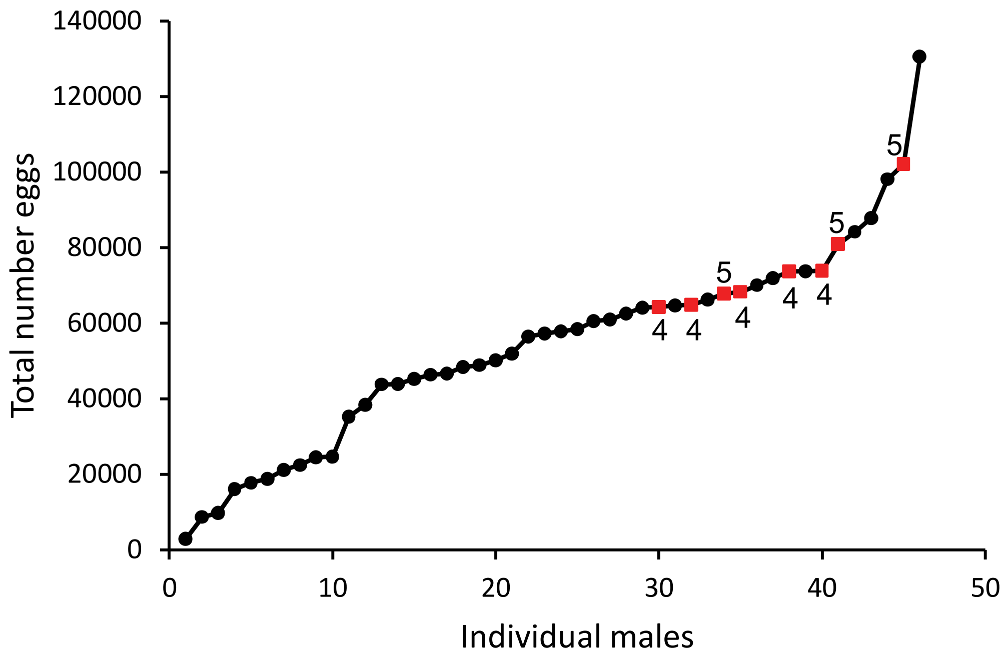

Figure S4. Total number of eggs present on 47 nests guarded by *Pomacentrus amboinensis* males over a 6-week period from the Lizard island fringing reef. Red squares are the total numbers of eggs for males that were found to have *P. amboinensis* eggs in their guts when collected at the end of the 6-week period. Numbers represent the relative gut fullness (0 empty to 5 totally full).

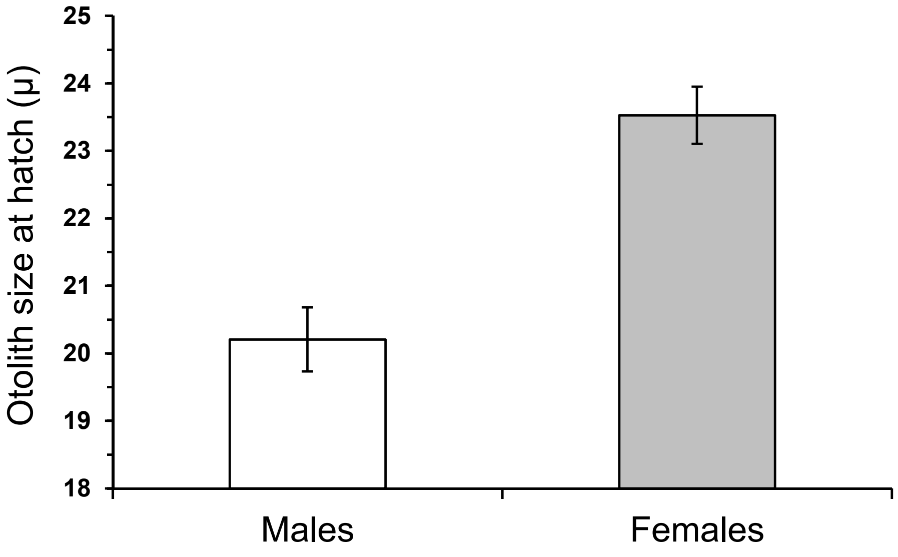

Figure S5. Mean maximum width (± SE) of sagittal otoliths at hatching for males *Pomacentrus amboinensis*, and females that have changed sex to become males. N = 15 females and 12 males).
